# Childbearing in adolescence: intergenerational *dejà*-*vu*? Evidence from a Brazilian birth cohort

**DOI:** 10.1186/1471-2393-13-149

**Published:** 2013-07-15

**Authors:** Alexandre Archanjo Ferraro, Viviane Cunha Cardoso, Aline Pires Barbosa, Antônio Augusto Moura Da Silva, Carlos Augusto Faria, Valdinar Souza De Ribeiro, Heloisa Bettiol, Marco Antonio Barbieri

**Affiliations:** 1 Faculty of Medicine, University of São Paulo, Avenida Dr. Enéas Carvalho de Aguiar 647, São Paulo, Brazil; 2 Ribeirão Preto School of Medicine, University of São Paulo, Avenida Bandeirantes 3900, Ribeirão Preto, São Paulo, Brazil; 3 Departament of Public Health, Federal University of Maranhão, rua Barão de Itapary 155, São Luís, Maranhão, Brazil; 4 Faculty of Medicine, Federal Fluminense University, Rua Marques do Paraná 303, Niterói, Rio de Janeiro, Brazil; 5 Departament of Medicine III, Federal University of Maranhão, Rua dos Prazeres 215, São Luís, Maranhão, Brazil

**Keywords:** Adolescent pregnancy, Socioeconomic predictors, Birth cohort

## Abstract

**Background:**

Pregnancy in adolescence tends to repeat over generations. This event has been little studied in middle and low-income societies undergoing a rapid epidemiological transition. To assess this association it is important to adjust for socioeconomic conditions at different points in lifetime. Therefore, the aim of this study is to analyze the independent effect of adolescent childbearing in a generation on its recurrence in the subsequent generation, after adjusting for socioeconomic status at different points in life.

**Methods:**

The study was conducted on a prospective cohort of singleton liveborn females from the city of Ribeirão Preto, Brazil, evaluated in 1978/79, and their daughters assessed in 2002/04. A total of 1059 mother-daughter pairs were evaluated. The women who had their first childbirth before 20 years of age were considered to be adolescent mothers. The risk of childbearing in adolescence for the daughter was modeled as a function of the occurrence of teenage childbearing in her mother, after adjustment for socio-demographic variables in a Poisson regression model.

**Results:**

The rate of childbearing during adolescence was 31.4% in 1978/79 and 17.1% in 2002/04. Among the daughters of the 1st generation adolescent mothers, this rate was 26.7%, as opposed to 12.7% among the daughters of non adolescent mothers. After adjustments the risk of adolescent childbearing for the 2nd generation was 35% higher for women whose mothers had been pregnant during adolescence – RR = 1.35 (95% CI 1.04-1.74).

**Conclusion:**

Adolescent childbearing in the 1st generation was a predictor of adolescent childbearing in the 2nd, regardless of socioeconomic factors determined at different points in lifetime.

## Background

According to the data of the Brazilian Health Ministry, during the last decade there has been a fall in the percentage of liveborn babies delivered by adolescent mothers among the total number of births from 23.3% to 19.3%. In absolute terms, this number is still high, corresponding to more than 550,000 births in 2010. However, among the less privileged classes, this rate showed a discrete increase [1]. These pregnancies are associated with both a higher obstetrical [2] and pediatric [3-6] risk.

Children of adolescent mothers show poorer eductional achievement, and this effect is more important for boys compared to girls [7]. In addition they have a higher probability of not reaching self-sufficiency standards in adult life, such as a higher educational level and financial independence, and of becoming adolescent parents themselves with the risks and personal costs associated with early parenthood [8-10].

Studies have suggested that pregnancy in adolescence is related to social risk situations. Usually it is not desired, it was not planned and it is considered to be a product of lack of information [11,12] and of an unfavorable socioeconomic situation [13]. Young mothers are more likely to be non-partnered or cohabiting rather than being married to the father of the child [14]. Furthermore, its occurrence may be associated with low schooling [15-17] and with neglect or abuse during childhood [18].

The challenge faced by studies that intend to identify predictors of adolescent pregnancy is an appropriate adjustment for the confounding effect of socioeconomic conditions. These can actually change during the life cycle, and more clearly so in societies in epidemiological transition. Repeated measurements over time are necessary for an appropriate adjustment of the socioeconomic conditions.

Daughters of adolescent mothers are at higher risk of becoming adolescent mothers [19-21]. It is not clear whether the elevated risk of recurrence of adolescent pregnancy in the second generation continues to be present after the control of socioeconomic confounding factors at different points in life. This phenomenon has been little studied in developing countries where the rates of adolescent pregnancy are higher.

The objective of the present study was to analyze the association between childbearing in adolescence and its occurrence in the next generation, after adjusting for socioeconomic and biological factors at different points in the lifetime in a cohort of women born in the city of Ribeirão Preto, in Southeastern Brazil.

## Methods

### Study design and reference population

**Figure 1:**
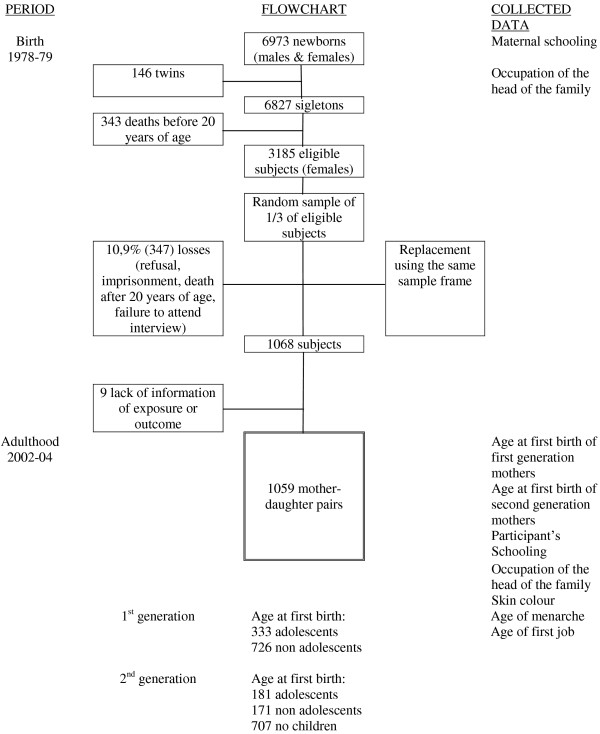
Population and sampling.

This was a prospective observational study of the cohort type involving liveborns in the city of Ribeirão Preto, evaluated from June 1, 1978 to May 31, 1979, with the objective of studying perinatal factors related to baby health at birth and to infant mortality [22]. The reference population consisted of women residing in the same municipality who had delivered a live newborn at all 8 maternity hospitals (1st generation) and their daughters evaluated in 2002/04 at 23–25 years of age (2nd generation). In this second phase, we evaluated factors that, operating at the beginning of life and during the subsequent years, are related to adult health [23]. Newborn whose mothers did not reside in the city were excluded, with a total of 6973 liveborns being left, 6827 singletons and 146 twins. Twins and 343 subjects who died before 20 years of age were excluded, with 6484 subjects (males and females) being left. One of each 3 subjects belonging to the same geographic area was invited to participate in the follow-up study. In the traced group, losses to follow-up (N=705) occurred because of refusal to participate, imprisonment, death after 20 years of age, or failure to attend the interview. Losses were replaced using the same sampling frame, resulting in 2063 young adults [23], corresponding to 31.8% of the eligible population, 1068 of them being women. A total of 1059 mother-daughter pairs were available for the study, with information being available about age at first childbirth of the daughter, if this was the case, and age at first childbirth of the mother (Figure 1). Women who delivered their first child before 20 years of age were considered to be adolescent mothers. The mothers of the original study of the birth cohort (1978/79) are referred to as “1st generation” and their daughters evaluated during adult age (2002/04) as “2nd generation”.

A sample size of 1,059 has a 90% power to detect an RR of 1.7, assuming the event has a 10% prevalence in the control group, with a 5% probability of type I error.

The study was approved by the Research Ethics Committee of the University Hospital, Faculty of Medicine of Ribeirão Preto, University of São Paulo (protocol 7606/99) and all subjects gave written informed consent to participate. Details of the study methodology are available in other publications [22-24].

### Instruments and variables of the study

We used a questionnaire with sociodemographic and biological information applied to 1059 2nd generation women, in addition to the questionnaire applied to their mothers in 1978/79 (1st generation). The following variables concerning the 1st generation were recorded: age at first childbirth in complete years, schooling in years of study (up to 4, 5 to 8, 9 or more); occupation of family head based on the International Classification of Occupation [25] (non-manual, skilled and semi-skilled manual, unskilled manual and outside the economically active population - EAP). For the 2nd generation the following information was recorded: age at first childbirth in complete years, when it occurred, schooling in years of study (up to 8, 9 or more) and occupation of family head.

Information on ethnicity was collected as the National Census does, i.e., self-reported skin colour and was categorized as white and non-white. Brazil has the largest African origin population outside Africa and presents a substantial race inequality. Official data suggest that in the period this study was done non-white male workers earned 63% less income than white ones [26]. This gap is mainly due to the advantages of whites in human capital, both own and parental education [27].

In 2002–04 retrospective information was collected regarding the adolescence period of the second generation: age at menarche (<12, 12, >12 years) and age at first job in complete years (<14, 14 to 17, > 17 or never worked).

According to the Brazilian Institute of Geography and Statistics in the 90’s most adolescent workers' (65%) came from families with an income of up to 1 minimum wage per capita [28]. The International Labour Organization reports as main causes for adolescent work the lack of access to education, poverty, specific vulnerability (low income or monoparental families and families with disabled people). Girls have a greater likelihood of receiving smaller wages, of starting to work earlier and of having the triple burden of a paid job, the domestic works and continuing to study [29]. For these reasons we chose to consider the age at first job as a proxy of the socioeconomic status during the adolescent years of the second generation, those of lower status tending to start earlier.

In 2002/04 schooling was categorized differently because in 1978–79 national compulsory schooling was 4 years and in 2002–04 it was already 8 years. In this sense lower categories in both generations represent the compulsory schooling. The highest category (9 or more years) was identical in both periods and it was chosen as the reference one.

### Data processing and analysis

Childbearing rates during adolescence were calculated for the two generations. Poisson regression models with robust estimate of variance were constructed to determine the independent effect of adolescent childbearing in the 1st generation on the outcome (adolescent childbearing in the 2nd generation). The incidence rate ratios (IRR) and their respective 95% confidence intervals (95% CI) were calculated.

The collection of two socioeconomic variables, occupation and schooling, was repeated at an interval of 23/24 years.

The multivariable model was adjusted for the socioeconomic variables of 1978/79 and 2002/04 as well as for age at menarche and age at first job. The linearity test was applied to investigate the presence of a gradient in the effect of exposure.

The STATA 10.1® statistical package was used for all analyses and the level of significance was set at 5%.

## Results

The characteristics of those women who completed the study and of those that have been lost are presented in Table 1. Lost subjects tended to come from less social advantage families.

**Table 1:** **Composition of the characteristics of the cohort at birth** (**1978**/**79**) **with those followed up in adulthood** (**2002**/**2004**)

**Variables**	**Initial population at birth 1978**/**79 n (%)**	**Individuals not interviewed in 2002**/**2004 n (%)**	**Individuals interviewed in 2002**/**2004 n (%)**	**P-value**
**Occupation of family head**				0.013
Non-manual	517 (16.2)	340 (16.0)	177 (16.7)	
Skilled and semi-skilled manual	1835 (57.6)	1192 (56.1)	643 (60.7)	
Unskilled manual or unemployed	725 (22.8)	518 (24.4)	207 (19.5)	
Missing	108 (3.4)	76 (3.6)	32 (3.0)	
**Mother**’**s schooling** (**years**)				< 0.001
Up to 4	1577 (49.5)	1100 (51.7)	477 (45.0)	
5 to 8	786 (24.7)	491 (23.1)	295 (27.9)	
9 and more	738 (23.2)	468 (22.0)	270 (25.5)	
Missing	84 (2.6)	67 (3.2)	17 (1.6)	
**Total**	3185	2126	1059	

The rate of childbearing in adolescence was 31.4% for the 1st generation and 17.1% for the 2nd generation (Table 2).

**Table 2:** **Maternal age at 1st childbirth among 1st and 2nd generation women in Ribeirão Preto**, **1978**/**79 and 2002**/**04** - **N (%)**

**1st generation**	**2nd generation**			**Total**
	**Up to 19 years**	**20 to 25 years**	**No children**	
Up to19 years	89 (26.7)	70 (21.0)	174 (52.3)	333 (100)
20 to 25 years	75 (14.2)	82 (15.6)	370 (70.2)	527 (100)
≥ 26 years	17 (8.5)	19 (9.6)	163 (81.9)	199 (100)
Total	181 (17.1)	171 (16.2)	707 (66.8)	1059 (100)

χ^2^ for trend = 7.46 p< 0.001; Chi-square for trend was calculated across categories of the second generation mothers by categories of first generation mothers.

Daughters of adolescent mothers had a higher proportion of childbearing in adolescence (26.7%) than daughters of older mothers. The lower proportion of “non-mothers” at the age of 23–25 years among the daughters of adolescent mothers (52.3%) suggested that these daughters had an earlier beginning of their reproductive phase (Table 2).

Bivariate analysis of the socioeconomic and biological factors associated with outcome is presented in Table 3. Daughters of adolescent mothers had a 2.11 times higher risk to also become adolescent mothers. At the time of birth of the participants and at the time when they became mothers, the type of work of the family head characterized as “unskilled manual and outside the EAP” was the one most associated with the outcome (25.1% and 24.7%). Childbearing during adolescence occurred more frequently among 1st and 2nd generation women with the lowest educational level, with a statistically higher prevalence in the latter group: 23.9% and 54.8%. While in the 1st generation 772 (74.7%) women had less than 9 years of study, in the 2nd generation they were only 155 (14.6%). Teenage childbearing was also higher among mothers with earlier menarche (20.2%), who entered the job market earlier (29.6%), and for non-white women (24.8%).

**Table 3:** Bivariate analysis of factors associated with adolescent pregnancy in the second generation

**Variables**	**Not adolescent mothers and no children**	**Adolescent mothers**	**RR**	**95% CI**	**p †**
**First generation**					
*Adolescent mother*					<*0*.*001*
Yes	244 (73.3)	89 (26.7)	2.11	1.62–2.74	
No	634 (87.3)	92 (12.7)	1.00		
*Occupation of family head*					<*0*.*001*
Non-manual	164 (92.7)	13 (7.3)	1.00		
Skilled and semi-skilled manual	532 (82.7)	111 (17.3)	2.35	1.36–4.07	
Unskilled manual or unemployed	155 (74.9)	52 (25.1)	3.42	1.93–6.07	
*Mother*’*s schooling*					<*0*.*001*
Up to 4 years	363 (76.1)	114 (23.9)	4.61		
5 to 8 years	244 (82.7)	51 (17.3)	3.33	2.70–7.87	
9 years and more	256 (94.8)	14 (5.2)	1.00	1.89–5.88	
**Second generation**					
*Occupation of family head*					<*0*.*001*
Non-manual	312 (91.5)	29 (8.5)	1.00		
Skilled and semi-skilled manual	446 (80.1)	111 (19.9)	2.34	1.59–3.45	
Unskilled manual or unemployed	122 (75.3)	40 (24.7)	2.90	1.87–4.51	
*Participant*’*s schooling*					<*0*.*001*
Up to 8 years	70 (45.2)	85 (54.8)	5.18	4.09–6.57	
9 years and more	811 (89.4)	96 (10.6)	1.00		
*Age at menarche*					*0*.*085*
< 12 years	233 (79.8)	59 (20.2)	1.45		
12 years	287 (82.0)	63 (18.0)	1.29	1.04–2.02	
> 12 years	351 (86.0)	57 (14.0)	1.00	0.93–1.79	
*Age at first job*					<*0*.*001*
< 14 years	131 (70.4)	55 (29.6)	1.00		
14 to 17 years	388 (80.7)	93 (19.3)	0.65	0.49–0.87	
> 17 years or never worked	362 (91.7)	33 (8.3)	0.28	0.19-0.42	
*Referred skin color*					<*0*.*001*
White	628 (86.0)	102 (14.0)	1.00		
Non-white	239 (75.2)	79 (24.8)	1.78	1.37–2.31	
Total	881	181			

† Wald test for the dichotomous variables and linearity test for the categorical ordinal variables; NOTE: numbers may not add up to total because of missing values.

Ribeirão Preto, 2002/04.

When all explanatory variables were adjusted simultaneously, the effect of childbearing in the 1st generation was 35% higher (RR=1.35, 95% CI 1.04-1.74); age at menarche, age at first job and schooling of the 2nd generation continued to be significantly associated with adolescent childbearing in the 2nd generation (Table 4).

**Table 4:** Multivariable analysis of factors associated with childbearing in adolescence in the second generation

		**All**	
	**RR**	**95% CI**	**p**
**First generation**			
*Childbearing in adolescence*			0.023
No	1.00	1.04-1.74	
Yes	1.35		
*Schooling* (*years*)			0.154
9 or +	1.00	0.94-3.28	
5 to 8	1.76	0.99-3.55	
Up to 4	1.88		
*Occupation of family head*			0.135
Non-manual	1.00	0.98-2.17	
Skilled and semi-skilled manual	1.46	0.98-2.42	
Unskilled manual or unemployed	1.54		
**Second generation**			
*Age at menarche* (*years*)			0.040
>12	1.00	0.86-1.62	
12	1.18	1.09-2.07	
<12	1.50		
*Age at first job* (*years*)			0.018
<14	1.00	0.63-1.11	
14–17	0.84	0.36-0.83	
>17	0.55		
*Schooling* (*years*)			<0.001
9 or +	1.00	2.61-4.48	
Up to 8	3.42		
*Occupation of family heaD*			0.966
Non-manual	1.00	0.62-1.79	
Skilled and semi-skilled manual	1.05	0.61-1.89	
Unskilled manual or unemployed	1.08		
*Referred skin color*			0.474
White	1.00	0.85-1.42	
Non-white	1.10		

*RR* relative risk, *CI* confidence interval “p” refers to the Wald test for the dichotomous variables and to the linearity test for the ordinal variables.

Ribeirão Preto, 2002/04.

## Discussion

In the present study conducted on a population-based sample, a lower childbearing rate in adolescence was observed in the 2nd generation compared to the 1st. There was an association of the occurrence of adolescent childbearing among 2nd generation daughters with the occurrence of adolescent childbearing among the 1st generation mothers even after adjustment for socioeconomic conditions at different points in life. A higher risk of childbearing during adolescence in the 2nd generation was also observed among women with earlier age at menarche, earlier access to the job market and lower schooling.

Many studies have looked for risk factors for adolescent pregnancy [30-32]. However the effect of early childbearing on the next generation is rarely considered. In those studies where this variable was included in the multivariate analyzes results are conflicting. In Australia adolescent pregnancy was not independently associated with whether the girl’s mother had been a teenage mother [33]. On the other hand in Finland, having a young mother had an independent association with becoming a teenage mother [34].

The important reduction in adolescent childbearing rate between generations (from 31.4% to 17.1%) occurred in parallel to improved socioeconomic situation: the percentage of mothers with low schooling was reduced from 74.1% to 14.6% and the percentage of family heads who were unskilled manual workers was reduced from 20.2% to 15.3%. This demonstrates that we are witnessing a society in socioeconomic transition, with improved schooling and qualification, suggesting that improved living conditions contributed to the reduction of pregnancy rate during adolescence. A fall in the annual rate of adolescent pregnancy occurred in Brazil from 2000 to 2009. According to the Health Ministry, the main explanation for the current decline is the investment in campaigns directed at adolescents and the expanded access to family planning [1]. However, at the time when this birth cohort was developed (1980’s and 1990’s) there was a marked increase in access to school in the Brazilian population, as confirmed by the data from the present sample. This was possibly one of the factors that contributed to the fall in childbearing rate during adolescence observed for the 2nd generation.

The risk of recurrence of adolescent pregnancy among daughters of adolescent mothers is normally attributed to socioeconomic factors [13]. The question is whether this event is more influenced by the socioeconomic situation at the beginning of life or at the time of pregnancy. Societies undergoing a rapid epidemiological and economic transition are ideal for the evaluation of this aspect. In the present study, the size of the effects of occupation of family head was similar in 1978/79 and 2002/04 after adjustment. In contrast, schooling had a more important effect in the second generation. Even though they are intimately associated, occupation and schooling measure different aspects of the socioeconomic factor.

In addition, the association between schooling and childbearing during adolescence can be explained by reverse causality: by becoming pregnant at an early age, girls may tend to drop out of school. In this case early age at childbearing could have been the cause of early school drop-out rather than low schooling being a cause of childbearing during adolescence. However, since the cut-off point for low schooling in the 2nd generation was eight years, and only 10 of these women became pregnant at 14 years of age or less (result not shown in a table), risk of reverse causality was minimized.

To confirm our choice to use ‘age at first job’ and ethinicity as a socioeconomic markers we performed an analyses which showed that in our sample among those who started working with less than 14 years of age, 92% were from families whose heads were engaged in manual occupations. And among those of non-white skin colour, 93% were from families whose heads were engaged in manual occupations (both with p<0.001 - not presented within the manuscript).

These socioeconomic markers did not fully explain the recurrence of childbearing during adolescence.

In the search for an additional explanatory factor, this time a biological one, we analyzed age at menarche. The literature shows that there is a positive association between mothers and daughters regarding age at menarche [20]. An earlier menarche means an earlier development of secondary sex traits and of reproductive capacity. Thus, a girl will start sporadic or steady dating relationships at an earlier time [35,36], a fact that, in turn, also favors an early start of sexual activity, which is not always accompanied by adequate means of prevention of pregnancy [37,38].

Perhaps the explanation for the independent association detected here may also be related to mechanisms linked to cultural variables, behaviors and values not included in the present study, but indirectly and partially measured based on the socioeconomic variables. Daughters of mothers who became pregnant during adolescence may consider this event to be less serious and risky compared to the general population and therefore they use less prevention [5]. In fact, some studies have shown that pregnancy was desired by the adolescent girl [39] and that pregnancy was not a negative experience for most of them [4,13].

A limitation of the study is that there was selective attrition. Those women whose family heads were engaged in unskilled occupations or were outside the economically active population and those with up to 4 years of schooling had lower follow-up rates than their counterparts. Since the better off were overrepresented in our final sample it is possible that estimates of the effect of childbearing in adolescence were underestimated, because childbearing rates are higher for the low SES groups. Another limitation was the lack of information about age at first sexual relation, use of contraceptive methods, number of partners, religion, and whether or not the pregnancy had been planned. This information would have permitted us to test some of the hypotheses mentioned. Furthermore, it is possible that residual confounding would be present whereby the effects of socioeconomic status may not have been fully accounted for by the measures that were available.

The strong points of the present study were the use of a population-based sample, having consistent socioeconomic information at three points in life and not having detected studies with similar design and analysis in the literature.

## Conclusion

A history of adolescent childbearing in the 1st generation seems to be a predictor of adolescent childbearing in the 2nd generation regardless of socioeconomic factors determined at different points in life.

Adolescent pregnancy is a worldwide problem and understanding its predictors is an important task. The higher risk of getting pregnant during adolescence that daughters of pregnant mothers have is difficult to be assessed independently since it is highly associated with social demographic variables.

We found an independent effect of being an adolescent mother on the occurrence of the same outcome in the next generation, even after multiple adjustments. The present study design allows us to consider this evidence reasonably robust.

## Abbreviation

EAP: Economically active population.

## Competing interests

The authors declare that they have no competing interests.

## Authors’ contributions

Study design MAB, HB, AAMS, Data collection APB, VSR, Analysis, and interpretation of data AAF, MAB, HB, AAMS, CAF, VVC, Writing of the report AAF, CAF, MAB, HB, AAMS, VVC, Decision to submit the manuscript for publication AAF, CAF, MAB, HB, AAMS, VVC. All authors read and approved the final manuscript.

## Pre-publication history

The pre-publication history for this paper can be accessed here:

http://www.biomedcentral.com/1471-2393/13/149/prepub
